# P02.63. Efficacy of an 8-week online mindfulness stress management program in a corporate call center

**DOI:** 10.1186/1472-6882-12-S1-P119

**Published:** 2012-06-12

**Authors:** D Allexandre, A Neuman, J Hunter, T Morledge, M Roizen

**Affiliations:** 1 Cleveland Clinic, Cleveland, USA; 2 Revati Wellness, Cleveland, USA

## Purpose

Stress is inherent in our society and results in great suffering, increased healthcare cost, and impaired work performance. An online program can offer a cost effective and practical solution that can help cope better with stress. In this study, we evaluated the effectiveness of an 8-week online mindfulness stress reduction (OSR) program in reducing work related stress and burnout and in improving well-being in a corporate call center. We also evaluated whether 1-hour weekly group practice and experience sharing at the workplace would improve program retention and engagement.

## Methods

161 participants were randomized either to wait-list control (CTL, N=37), OSR (N=54) or OSR and weekly group meetings (OSR+grp, N=70). The Perceived Stress Scale, Maslach Burnout Inventory (professional efficacy and exhaustion subscales), Mindful Attention Awareness Scale and SF36 (emotional well-being and role functioning subscales) were administered at baseline, post intervention and at 8-week follow-up.

## Results

We observed overall a greater post intervention decrease in stress and exhaustion and increase in mindfulness, emotional well-being and role functioning in the OSR+grp (cohen d=1.3, 0.8, 0.6, 1.4 respectively) compared to OSR (d=1, 0.4, 0.5, 0.8) and wait-list control (d<0.4). This overall improvement was also maintained at follow-up for most measures. The improvement was significantly greater for the OSR compared to CTL for stress and emotional well-being, and for OSR+grp compared to CTL for all outcomes except for professional efficacy. OSR+grp improved significantly more than OSR for stress, emotional well-being and emotional role functioning. Weekly group practice significantly increased program engagement and reduced dropout rate (13% for OSR+grp compared to 55% for OSR).

## Conclusion

An online mindfulness stress management program when combined with weekly group practice can offer a practical and cost-effective approach to decrease stress and burnout and improve mindfulness and well-being at the workplace.

